# Mouse Stbd1 is *N*-myristoylated and affects ER–mitochondria association and mitochondrial morphology

**DOI:** 10.1242/jcs.195263

**Published:** 2017-03-01

**Authors:** Anthi Demetriadou, Julia Morales-Sanfrutos, Marianna Nearchou, Otto Baba, Kyriacos Kyriacou, Edward W. Tate, Anthi Drousiotou, Petros P. Petrou

**Affiliations:** 1 Department of Biochemical Genetics, The Cyprus Institute of Neurology and Genetics, P. O. Box 23462, Nicosia 1683, Cyprus; 2 The Cyprus School of Molecular Medicine, P. O. Box 23462, Nicosia 1683, Cyprus; 3 Department of Chemistry, Imperial College London, Exhibition Road, London SW7 2AZ, UK; 4 Department of Electron Microscopy / Molecular Pathology, The Cyprus Institute of Neurology and Genetics, P. O. Box 23462, Nicosia 1683, Cyprus; 5 Oral and Maxillofacial Anatomy, Faculty of Dentistry, Tokushima University, Tokushima 770-8504, Japan

**Keywords:** Stbd1, Glycogen, Endoplasmic reticulum, Mitochondria, *N*-myristoylation, Mitochondria-associated membranes, Organized smooth endoplasmic reticulum

## Abstract

Starch binding domain-containing protein 1 (Stbd1) is a carbohydrate-binding protein that has been proposed to be a selective autophagy receptor for glycogen. Here, we show that mouse Stbd1 is a transmembrane endoplasmic reticulum (ER)-resident protein with the capacity to induce the formation of organized ER structures in HeLa cells. In addition to bulk ER, Stbd1 was found to localize to mitochondria-associated membranes (MAMs), which represent regions of close apposition between the ER and mitochondria. We demonstrate that *N*-myristoylation and binding of Stbd1 to glycogen act as major determinants of its subcellular targeting. Moreover, overexpression of non-myristoylated Stbd1 enhanced the association between ER and mitochondria, and further induced prominent mitochondrial fragmentation and clustering. Conversely, shRNA-mediated Stbd1 silencing resulted in an increase in the spacing between ER and mitochondria, and an altered morphology of the mitochondrial network, suggesting elevated fusion and interconnectivity of mitochondria. Our data unravel the molecular mechanism underlying Stbd1 subcellular targeting, support and expand its proposed function as a selective autophagy receptor for glycogen and uncover a new role for the protein in the physical association between ER and mitochondria.

## Abstract (precis)

**Highlighted Article:** The glycogen-binding protein Stbd1 is *N*-myristoylated and targeted to ER–mitochondria contact sites. Stbd1 loss- or gain-of-function affects ER–mitochondria association and mitochondrial morphology.

## INTRODUCTION

Starch binding domain-containing protein 1 (Stbd1; also known as genethonin-1, GENX-3414) was originally identified in a large-scale differential expression screen for genes displaying specific or increased expression in human skeletal muscle (Bouju et al., 1998). The function of the protein remained largely unknown until it was strongly implicated in the metabolism and cellular trafficking of glycogen (Jiang et al., 2010). Both in humans and mice, *Stbd1* was shown to be predominantly expressed in muscle and liver, the main tissues of glycogen synthesis and storage (Bouju et al., 1998; Jiang et al., 2010).

Stbd1 harbours an N-terminal hydrophobic region and a C-terminal carbohydrate-binding module (CBM20), which are highly conserved in mammalian species, as well as a less-conserved putative leucine-zipper motif. Through its N-terminal region, Stbd1 was suggested to associate with the membranes of the endoplasmic reticulum (ER) (Jiang et al., 2010; Zhu et al., 2014). The CBM20 domain, on the other hand, was shown to mediate binding to glycogen and related carbohydrates (amylose, amylopectin and polyglucosans) (Jiang et al., 2010; Zhu et al., 2014). In addition, the same domain was identified as being important for the dimerization of the protein (Jiang et al., 2010), as well as for its stability and interaction with other glycogen-related proteins such as laforin, glycogen synthase and glycogen-debranching enzyme (Zhu et al., 2014). No specific function has so far been assigned to the leucine-zipper domain.

When overexpressed in cultured cells, human Stbd1 was found to concentrate to prominent rounded perinuclear structures which coincided with ER markers and large glycogen deposits (Jiang et al., 2010). Localization of Stbd1 to these structures required the presence of the N-terminal hydrophobic region since deletion of the first 24 amino acids resulted in a diffused cytoplasmic distribution of the protein (Jiang et al., 2010).

A link between Stbd1 and autophagy was suggested based on the identification of an Atg8-family interacting motif (AIM), which is highly conserved in mammals, through which Stbd1 was shown to interact with Gabarapl1, a member of the Atg8 family of autophagy proteins (Jiang et al., 2011). Based on this finding and in conjunction with its capacity to bind glycogen, Stbd1 was proposed to be a selective autophagy receptor for glycogen, mediating its trafficking to lysosomes by means of an autophagy-like process. For this proposed mechanism, the term ‘glycophagy’ was coined (Jiang et al., 2011). Based on these findings, Stbd1 was considered an attractive target for therapy for Pompe disease (glycogen storage disease type II; OMIM #232300), a severe metabolic myopathy characterized by the intralysosomal accumulation of glycogen due to the inherited deficiency of the enzyme acid α-glucosidase (GAA) (Chen et al., 2009). This hypothesis was addressed by means of a Stbd1 knockdown approach in *Gaa^−/−^* mice. Despite a reduction in *Stbd1* expression levels by 23–28% in skeletal and cardiac muscle, a decrease in the amount of accumulated glycogen in the affected tissues did not occur (Yi et al., 2013). However, a recent report showed that in *Stbd1*/*Gaa* double knockout mice, glycogen storage is reduced in the liver but not muscle, supporting a role for Stbd1 in lysosomal glycogen transport in the liver (Sun et al., 2016).

Here, we show that mouse Stbd1 is an ER-resident protein which also localizes to ER–mitochondria contact sites in HeLa cells. Furthermore, our findings indicate that Stbd1 induces the reorganization of the ER and the recruitment of glycogen to organized smooth ER (OSER) structures. We demonstrate that Stbd1 is *N*-myristoylated and that this lipid modification, together with the binding of glycogen to the protein, acts as a major determinant of its subcellular targeting. Finally, our findings reveal an unprecedented role of Stbd1 at ER–mitochondria interfaces since both its overexpression and functional knockdown affect the association between ER and mitochondria and the morphology of the mitochondrial network.

## RESULTS

### Stbd1 is an ER-resident protein and induces morphological changes in both the ER and mitochondrial network when overexpressed in HeLa cells

Mouse Stbd1 harbours a highly conserved N-terminal domain, which is predicted to be a transmembrane helix and a potential signal sequence using the TMHMM2.0 tool (http://www.cbs.dtu.dk/services/TMHMM/). The same algorithm predicted a topology for the protein compatible with a single-pass type III transmembrane protein (probability: 0.83). This N-terminal region has been previously suggested, in the human orthologue, to be important for its targeting to membranes including the ER membrane (Jiang et al., 2010).

We first addressed the question of whether Stbd1 is an ER-resident protein and examined the role of the N-terminal hydrophobic domain in its subcellular targeting. Deletion of the N-terminal region (amino acids 1–25) of mouse Stbd1 resulted in its cytosolic distribution (Fig. S1A–C), whereas a heterologous protein fused to the above hydrophobic domain [(1-25)–EGFP–Myc] displayed ER localization (Fig. S1D–F). Evaluation of the cell lysate and culture supernatant of HeLa cells transfected with mouse Stbd1 fused to a Myc epitope (Stbd1–Myc) or (1-25)–EGFP–Myc revealed that the above proteins were detected only in the cell lysate but not the culture supernatant (Fig. S1G). Taken together, the above findings suggest that Stbd1 is an ER-resident protein and that its N-terminal hydrophobic region functions as a signal-anchor sequence that is both necessary and sufficient for the targeting and retention of the protein to the ER membrane.

**Fig. 1.**
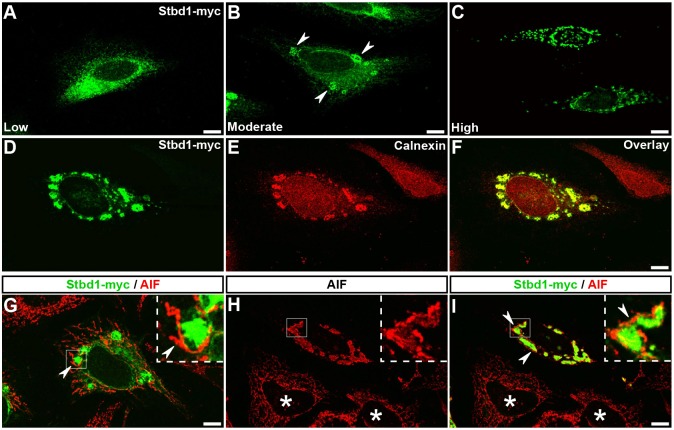
**Stbd1 localizes to the ER and induces morphological changes in both the ER and mitochondrial network.** (A–C) HeLa cells transiently transfected with Stbd1–Myc and immunostained for Myc displaying low (A), moderate (B) and high (C) levels of expression. (D­–F) HeLa cells transfected with Stbd1–Myc and stained for Myc (D) and calnexin (E); an overlay is shown in F. (G–I) Stbd1–Myc-transfected HeLa cells double stained for Myc and the mitochondrial marker AIF featuring moderate (G) and high (H,I) levels of expression. Inserts in G–I are high magnifications of the corresponding boxed areas. For all the above, representative images are shown. Arrowheads in B point to the rounded structures formed upon Stbd1 overexpression, and in G,I to mitochondria located at the periphery of ER structures. Asterisks in H,I indicate untransfected cells. Scale bars: 10 µm.

We next investigated the subcellular distribution of mouse Stbd1 by transient transfection of Stbd1–Myc in HeLa cells followed by immunofluorescence staining for the Myc epitope. Depending on the level of expression, variations in the immunofluorescence pattern were evident. At low expression levels, the protein displayed a uniform network-like staining (Fig. 1A), whereas in cells expressing higher levels of Stbd1, the formation of prominent rounded structures was apparent (Fig. 1B,C). At a moderate Stbd1 expression, these structures were large but fewer in number (Fig. 1B, arrowheads), but appeared smaller and more numerous at higher expression levels of the protein (Fig. 1C).

Staining of Stbd1–Myc-transfected cells for the ER marker calnexin identified these large structures as ER formations (Fig. 1D–F). A similar overlap was evident upon co-transfection with a vector expressing the ER marker Sec61β fused to mCherry (mCherry–Sec61β) (Fig. S2A). Nevertheless, no significant overlap was seen with lysosomes, visualized by means of LAMP1 staining (Fig. S2B). Assessment of a potential colocalization between Stbd1 and mitochondria labelled with an antibody against apoptosis-inducing factor (AIF) revealed the presence of mitochondria at the periphery of the large ER structures (Fig. 1G, arrowheads) but no significant overlap was apparent at low to moderate levels of Stbd1 expression (Fig. 1G). In contrast, in cells expressing high levels of Stbd1, the mitochondrial network displayed an altered morphology (Fig. 1H) and largely coincided with Stbd1 staining (Fig. 1I). Interestingly, the mitochondrial staining appeared more intense in the regions directly adjacent and surrounding the Stbd1-positive structures (Fig. 1I, arrowheads). Similar results were obtained using the outer mitochondrial membrane (OMM) protein TOM20 (also known as TOMM20) as a mitochondrial marker (Fig. S2C,D).

These findings indicate that Stbd1 overexpression induces morphological changes in both the ER and mitochondrial network, an effect that becomes more pronounced with increasing expression levels of the protein (Fig. S2E). The above may thus imply a role for Stbd1 in the physical association between ER and mitochondria.

### Stbd1 induces the formation of organized ER structures

**Fig. 2.**
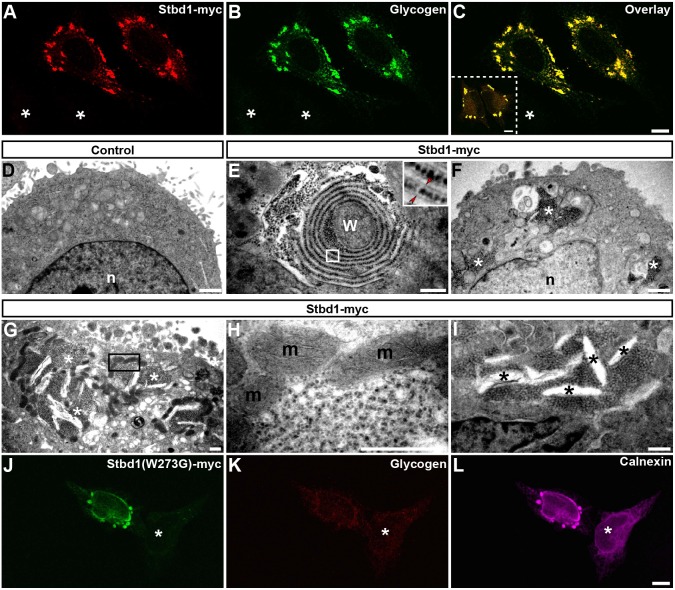
**Stbd1 induces the formation of organized ER structures.** (A–C) Representative images of Stbd1–Myc-transfected HeLa cells, immunostained for Myc (A) and glycogen (B); an overlay is shown in C. Glycogen strongly colocalizes with Stbd1 (thresholded Manders' coefficient, mean±s.e.m.: 0.848±0.019; *n*=10). (C, insert) Stbd1 not fused to a Myc epitope shows strong colocalization with glycogen in transfected cells, shown is the overlay of Stbd1 (green) and glycogen (red) immunofluorescence staining (thresholded Manders' coefficient, mean±s.e.m.: 0.853±0.004; *n*=7). (D–I) Representative transmission electron micrographs of untransfected control (D) or HeLa cells transfected with Stbd1–Myc (E–I) featuring OSER in the form of whorls (E) or stacked ER membranes (F–I), with electron-dense glycogen granules located between tethered membranes (insert in E, arrowheads; higher magnification of the boxed area). Clusters of mitochondria are seen at the periphery of ER structures (G,H). (H) A higher magnification of the boxed area indicated in G. (I) OSER structures containing regions of dilated ER (asterisks). Representative images of HeLa cells overexpressing Stbd1(W273G)–Myc (J) with compromised capacity to bind glycogen (K) displaying calnexin-positive OSER (L). Asterisks in A–C and J–L indicate untransfected cells, and in F,G, OSER. M, mitochondria; n, nucleus; w, whorl. Scale bars: 10 µm (A–C,J–L); 5 μm (D,F); 2 µm (E,G–I).

We sought to characterize the ER structures formed upon Stbd1 overexpression by means of transmission electron microscopy (TEM). For this, we took advantage of the previously reported property of Stbd1 to bind glycogen (Jiang et al., 2010). As revealed by immunofluorescence staining using a glycogen-specific antibody, HeLa cells overexpressing Stbd1–Myc displayed significant accumulation of glycogen, which strongly colocalized with Stbd1 (Fig. 2A–C), while glycogen was not detectable in untransfected cells (Fig. 2A–C, asterisks). A similar accumulation of glycogen in Stbd1-induced intracellular formations was observed upon overexpression of an untagged version of the protein (Fig. 2C, insert), suggesting that both the capacity of Stbd1 to induce the formation of ER structures and its ability to bind glycogen are intrinsic properties of the protein and not a Myc epitope-associated artefact. Thus, cells transiently transfected with Stbd1 can be easily identified and discriminated from untransfected cells at the electron microscope level by means of the massive accumulation of characteristic electron-dense glycogen granules. Furthermore, due to the very strong colocalization between glycogen and Stbd1, glycogen particles can indirectly serve as a marker of the subcellular distribution of Stbd1. TEM analysis revealed that in contrast to untransfected control cells (Fig. 2D), and consistent with the above-described immunofluorescence results, HeLa cells overexpressing Stbd1–Myc displayed rearranged ER membrane structures that were decorated with glycogen granules (Fig. 2E–I). These were apparent in the form of whorls (Fig. 2E) or stacked ER membranes (Fig. 2F–I). In these rearranged ER structures, glycogen granules did not coincide with ER membranes but were located in the regions between adjacent ER membranes (Fig. 2E, insert). Interestingly, and in agreement with the immunofluorescence staining of mitochondria in Stbd1-transfected cells (Fig. 1G), clusters of mitochondria were observed at the periphery of these stacked ER structures (Fig. 2G,H), which also often appeared to contain electron-lucent regions resembling dilated ER (Fig. 2I). The ER formations induced by the overexpression of Stbd1 were highly reminiscent of OSER structures. These have been shown to occur as a result of the overexpression of ER-resident transmembrane proteins able to undergo weak homotypic interactions (Snapp et al., 2003). Our results thus demonstrate that the rounded structures formed by the overexpression of Stbd1 in cultured cells represent regions of organized ER and identify Stbd1 as an OSER-inducing protein.

We addressed the question of whether Stbd1-induced OSER formation depends on the capacity of the protein to bind glycogen. A previous report identified a conserved tryptophan residue (W293) within the CBM20 domain of human Stbd1 as being important for its binding to glycogen (Jiang et al., 2010). We therefore engineered the corresponding mutation (W273G) in the mouse protein [Stbd1(W273G)–Myc] and first examined whether the glycogen-binding property of Stbd1 was abolished. Indeed, no accumulation of glycogen was evident in HeLa cells overexpressing Stbd1(W273G)–Myc (Fig. 2J,K). Nevertheless, despite its compromised ability to bind glycogen, Stbd1(W273G)–Myc appeared to be concentrated in rounded structures (Fig. 2J) that were also positive for calnexin (Fig. 2L), identifying them as OSER. The above suggests that tethering of ER membranes and OSER formation induced by Stbd1 overexpression is independent of glycogen binding.

**Fig. 3.**
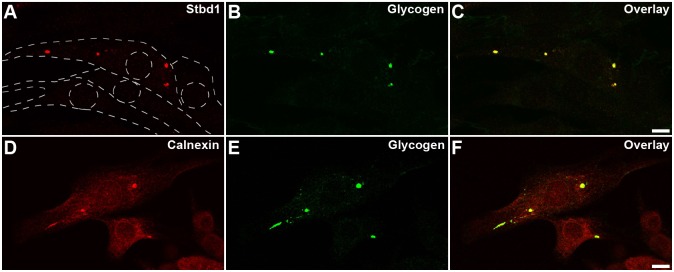
**Stbd1- and glycogen-positive ER structures are present endogenously in C2C12 cells.** (A–C) C2C12 mouse myoblast cells displaying colocalization of endogenous Stbd1 (A) and glycogen (B) in intracellular rounded structures; an overlay is shown in C. Staining for calnexin (D) and glycogen (E) (an overlay is shown in F) identifies these structures as ER formations. For the above, representative images are shown. Dotted lines in A indicate the cell periphery and nucleus. Scale bars: 10 μm.

We next enquired whether the aforementioned OSER structures are merely a consequence of Stbd1 overexpression or whether these also occur endogenously. For this, we monitored the subcellular localization of endogenous Stbd1 in a variety of cell lines. We found that in C2C12 mouse myoblasts grown under standard culturing conditions, endogenous Stbd1 colocalized with glycogen in intracellular rounded structures that were observed in ∼10% of cells (Fig. 3A–C). Moreover, these stained positive for calnexin, indicating that they represent ER formations (Fig. 3D–F). The above findings suggest that glycogen-containing Stbd1-positive organized ER regions are not solely induced by Stbd1 overexpression but are also formed endogenously.

### Stbd1 is *N*-myristoylated

Given the important role of the N-terminal hydrophobic region of Stbd1 for its targeting and retention to the ER membrane, we searched for potential post-translational modifications in this domain. An *in silico* search using the NMT-MYR-Predictor software (http://mendel.imp.ac.at/myristate/SUPLpredictor.htm) identified a reliable motif for *N*-myristoylation. *N*-myristoylation involves the addition of myristate, a saturated 14-carbon fatty acid, to an exposed N-terminal glycine residue through the action of an *N*-myristoyltransferase (NMT) enzyme (Farazi et al., 2001). In Stbd1, myristoylation is predicted to occur co-translationally at the glycine at position 2 (G2) following the removal of the initiator methionine.

**Fig. 4.**
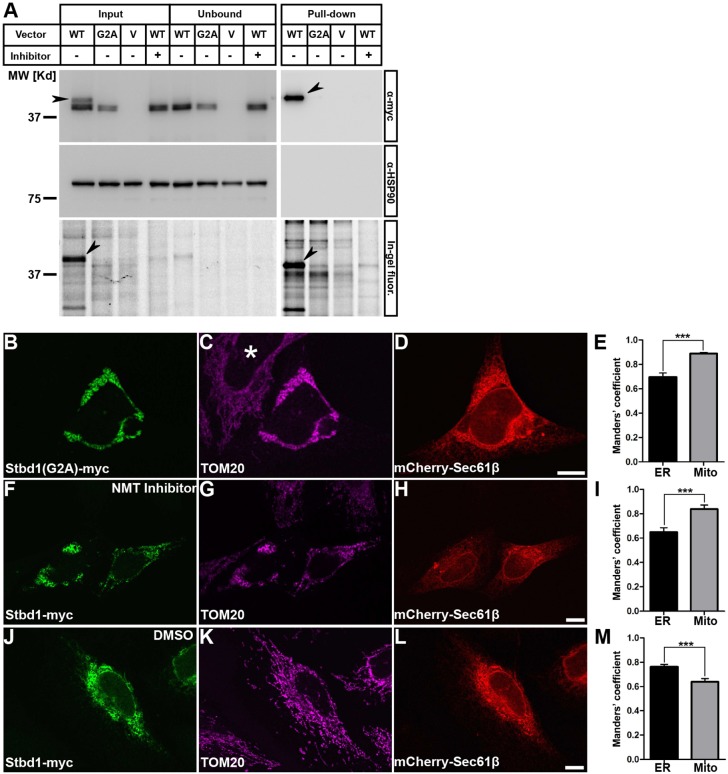
**Stbd1 is subjected to *N*-myristoylation, which affects its subcellular targeting.** (A) Metabolic labelling of HeLa cells transfected with either Stbd1–Myc (WT), Stbd1(G2A)–Myc (G2A) or the empty vector (V) with YnMyr, in the presence (+) or absence (−) of the NMT inhibitor DDD85646. Pulled-down proteins and supernatants before (‘Input’) and after pulldown (‘Unbound’) were analysed by western blotting (top and middle) and by in-gel fluorescence (bottom). Arrowheads indicate bands corresponding to *N*-myristoylated Stbd1. Equal loading and lack of unspecific proteins was confirmed by immunoblotting with an anti-HSP90 antibody. (B–D) Representative images of HeLa cells transfected with Stbd1(G2A)–Myc, immunostained for Myc (B) and TOM20 (C). ER is visualized by mCherry–Sec61β expression (D). Stbd1(G2A) strongly colocalizes with mitochondria and less with the ER (thresholded Manders' coefficient, mean±s.e.m.: mitochondria, 0.888±0.001; ER, 0.695±0.034; *n*=20) (E). (F–M) Representative images of HeLa cells transfected with wild-type Stbd1–Myc treated with 3.5 μM of the NMT inhibitor DDD85646 (F–H) or DMSO as vehicle control (J–L) and double stained for Myc (F,J) and TOM20 (G,K). The ER was stained by overexpressing mCherry–Sec61β (H,L). In cells treated with the inhibitor, Stbd1–Myc predominantly coincides with mitochondria and less with the ER (thresholded Manders' coefficient, mean±s.e.m.: mitochondria, 0.838±0.032; ER, 0.648±0.037; *n*=20) (I), whereas in control cells Stbd1–Myc displays stronger colocalization with the ER compared to mitochondria (thresholded Manders' coefficient, mean±s.e.m.: mitochondria, 0.639±0.027; ER, 0.763±0.018; *n*=13) (M). ***P<0.0001 (unpaired Student's *t*-test). Scale bars: 10 μm.

To evaluate whether Stbd1 is *N*-myristoylated, we first generated a Stbd1 protein variant unable to undergo *N*-myristoylation by replacing G2 with an alanine residue [Stbd1(G2A)–Myc]. Next, HeLa cells were transiently transfected with either wild-type Stbd1–Myc, the non-myristoylated Stbd1(G2A)–Myc mutant or the empty vector and were subjected to metabolic labelling using the alkyne-tagged myristate analogue YnMyr (Heal et al., 2008) in the absence or presence of the specific NMT inhibitor DDD85646 (Alibhai et al., 2013; Thinon et al., 2014). Cell lysates were subsequently incubated with azido-TAMRA-PEG-Biotin (AzTB) which is incorporated into YnMyr-labelled proteins via a click reaction and enables the detection of *N*-myristoylated proteins by means of in-gel fluorescence and western blotting. As assessed by western blotting, a band corresponding to the molecular mass of Stbd1 was detected in cells transfected with wild-type Stbd1 and the G2A mutant but not the empty vector in both the ‘input’ and ‘unbound’ protein sample (Fig. 4A, top left). Importantly, in cells transfected with wild-type Stbd1, a second band of slightly higher molecular mass was detected, which was not present in cells transfected with the G2A variant or the wild-type protein in the presence of the inhibitor (Fig. 4A, top left, arrowhead). This higher molecular mass band corresponds to *N*-myristoylated Stbd1 as it was detected in the ‘pull-down’ sample only in cells expressing wild-type Stbd1, but not cells expressing the G2A mutant or the wild-type protein in the presence of the inhibitor (Fig. 4A, top right), and occurs because the incorporation of the alkyne-azide complex causes a slight increase in the molecular mass of the protein. Similar results were obtained by in-gel fluorescence analysis, indicating the presence of fluorescent YnMyr-labelled protein only in cells transfected with wild-type Stbd1 in the absence of the inhibitor (Fig. 4A, bottom).

### *N*-myristoylation is a major determinant of Stbd1 subcellular localization between bulk ER and mitochondria-associated membranes

We investigated the role of *N*-myristoylation in the subcellular targeting of Stbd1. Interestingly, in transiently transfected HeLa cells, non-myristoylated Stbd1(G2A) appeared to strongly coincide with mitochondria and to a lesser extent with the ER (Fig. 4B–E). Overexpression of Stbd1(G2A) induced, in addition, a prominent change in the morphology of the mitochondrial network, characterized by clustering (Fig. 4C), as compared to that in control cells (Fig. 4C, asterisk and Fig. S3A). Upon closer examination, the immunofluorescence staining corresponding to Stbd1(G2A)–Myc and mitochondria was not completely overlapping (Fig. S3B–D), suggesting that Stbd1(G2A) is probably not directly targeted to mitochondria. Moreover, significant ER staining coinciding with regions of clustered mitochondria and Stbd1(G2A) immunofluorescence was evident in Stbd1(G2A)–Myc-transfected cells (Fig. 4B–D), suggesting that non-myristoylated Stbd1 may localize to ER regions that are in close apposition to mitochondria.

To confirm that the subcellular localization of Stbd1(G2A) is indeed due to the lack of *N*-myristoylation we evaluated the immunofluorescence staining pattern of wild-type Stbd1 in the presence of the NMT inhibitor DDD85646. Similar to the G2A mutant, Stbd1–Myc-transfected cells treated with the inhibitor displayed a stronger colocalization with clustered mitochondria than with the ER (Fig. 4F–I). In contrast, similarly transfected control cells treated with DMSO vehicle, exhibited the typical staining pattern of Stbd1 in the ER and OSER structures (Fig. 4J–M).

Taken together, the above findings demonstrate that *N*-myristoylation acts as a molecular switch and a major determinant of Stbd1 subcellular targeting. Incorporation of myristate appears to favour the retention of the protein in bulk ER, resulting in the formation of OSER, whereas its absence seems to promote targeting of the protein to ER regions that are in close proximity with mitochondria, known as mitochondria-associated membranes (MAMs).

**Fig. 5.**
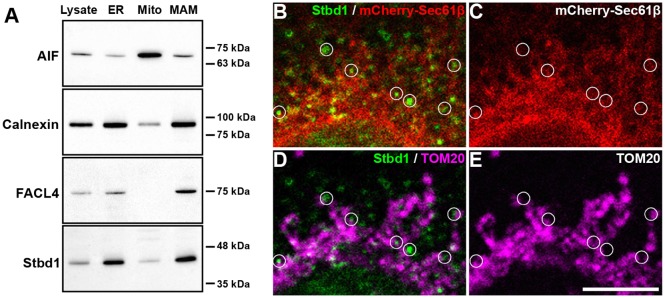
**Endogenous Stbd1 localizes to MAMs.** (A) Western blot on ER, mitochondrial (Mito) and MAM fractions (10 µg) obtained by subcellular fractionation of HeLa cells, probed with antibodies against Stbd1 and the marker proteins calnexin (localized in ER and MAMs), AIF (mitochondria) and FACL4 (enriched in MAMs). Total cell lysate (10 µg) was used as a positive control. (B–E) High-magnification representative images of HeLa cells stained for endogenous Stbd1 (green) (B,D) and mitochondria (magenta pseudocolor) (D,E). mCherrySec61β was used to stain the ER (B,C). Stbd1 puncta coinciding with the ER and localized in close proximity to mitochondria representing ER-mitochondria contact sites are indicated with circles. Scale bar: 5 μm.

To address the question of whether Stbd1 localizes to MAMs, we subjected HeLa cells to subcellular fractionation and evaluated the presence of endogenous Stbd1 in isolated fractions of ER, mitochondria and MAMs by western blotting. The following antibody markers were employed to assess the purity of the isolated fractions: AIF for mitochondria, calnexin for ER and MAMs and fatty acid CoA ligase 4 (FACL4, also known as ACSL4) which is widely used as a reliable marker protein for MAMs (Raturi and Simmen, 2013) (Fig. 5A). Using the above approach, endogenous Stbd1 was detected in both the ER and MAM fraction, but not the mitochondrial fraction, similar to calnexin (Fig. 5A), which is known to localize to both subcellular domains (Lynes et al., 2012). To confirm the subcellular fractionation results, we examined the localization of endogenous Stbd1 in HeLa cells by means of immunofluorescence staining. Stbd1 was detected in a punctate pattern in the ER (Fig. 5B,C) with several of the Stbd1-positive puncta being localized in close proximity to mitochondria (Fig. 5D,E). Taken together, the above data suggest that Stbd1 is endogenously targeted, in addition to bulk ER, to ER–mitochondria contact sites.

### Non-myristoylated Stbd1 affects mitochondrial morphology and ER-mitochondria contacts

**Fig. 6.**
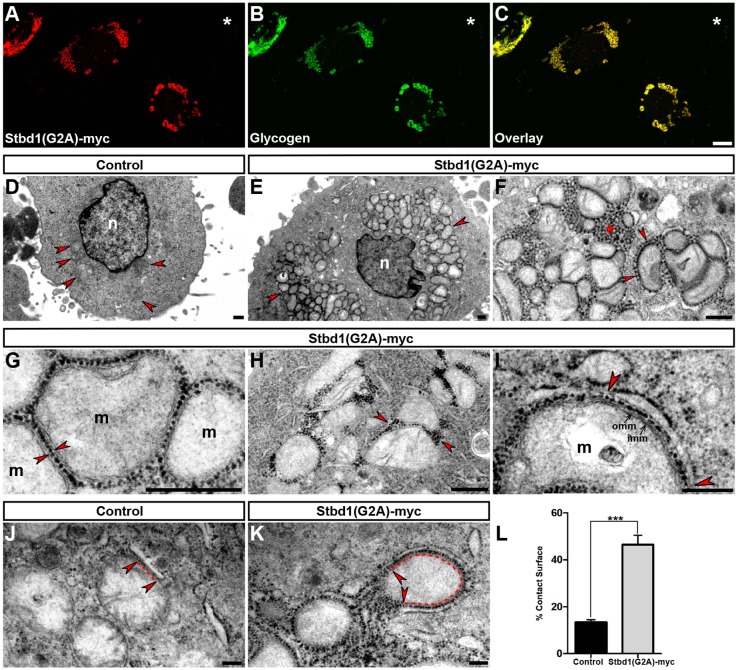
**Overexpression of non-myristoylated Stbd1 induces mitochondrial clustering and increases ER-mitochondria apposition.** (A–C) Representative images of HeLa cells transfected with Stbd1(G2A)–Myc stained for Myc (A) and glycogen (B); an overlay is shown in C. Glycogen strongly colocalizes with Stbd1(G2A) (thresholded Manders' coefficient, mean±s.e.m., 0.925±0.015; *n*=13). Asterisks in A–C indicate an untransfected cell. (D–I) Representative transmission electron micrographs of untransfected control (D, arrowheads indicate mitochondria) and Stbd1(G2A)–Myc-transfected HeLa cells displaying prominent clustering of mitochondria (E, arrowheads). (F) Glycogen particles reflecting the localization of the protein are found in regions of bulk ER (asterisk) and at ER–mitochondria contact sites (arrowheads). (G) In tightly clustered mitochondria, glycogen particles are present within the region confined by their OMM (arrowheads). (H) In smaller mitochondrial clusters, glycogen particles accumulate at the attachment sites (arrowheads) and are not uniformly distributed around mitochondria. (I) Glycogen granules are localized between the OMM and MAM (arrowheads). (J,K) As compared to untransfected controls (J), cells overexpressing Stbd1(G2A)–Myc display increased contact surface between ER and mitochondria (indicated by arrowheads and dotted line) (K). (L) Quantification of ER–mitochondria associations expressed as the percentage of the mitochondrial surface closely apposed to ER [mean±s.e.m., control, 13.37±1.1%; *n*=25; Stbd1(G2A)–Myc, 46.48±3.93%; *n*=29]. n, nucleus; m, mitochondria; omm, outer mitochondrial membrane; imm, inner mitochondrial membrane. ***P<0.0001 (unpaired Student's *t*-test). Scale bars: 10 µm (A–C); 2 µm (D–F,H); 1 µm (G,I–K).

To investigate the localization and the effects of the overexpression of non-myristoylated Stbd1 at the ultrastructural level, we employed again the property of the protein to bind glycogen, as evidenced by the significant glycogen accumulation in Stbd1(G2A)–Myc-transfected cells and its very strong colocalization with the non-myristoylated protein (Fig. 6A–C). As assessed by TEM, and in contrast to untransfected controls (Fig. 6D), HeLa cells overexpressing non-myristoylated Stbd1 displayed prominent mitochondrial clusters consisting of fragmented mitochondria (Fig. 6E, arrowheads). Evaluation of mitochondrial morphology parameters revealed a significant decrease in the total area and perimeter of the mitochondrial network in Stbd1(G2A)–Myc-expressing cells as compared to controls, consistent with clustering (Fig. S3E,F). However, due to the massive clustering, mitochondrial fragmentation could not be reliably quantified. In agreement with the immunofluorescence staining pattern (Fig. S3B–D), Stbd1(G2A)–Myc, as revealed by the distribution of glycogen particles, did not strictly localize to areas that were in the proximity of mitochondria but was also present in regions of bulk ER (Fig. 6F, asterisk). In large mitochondrial clusters, glycogen particles were found within the intervening spaces confined by the OMM (Fig. 6G, arrowheads). In contrast, in mitochondria that were not included in large clusters, glycogen granules were not uniformly distributed around mitochondria but appeared to accumulate at the attachment sites (Fig. 6H, arrowheads). Importantly, glycogen particles were evident between the OMM and the adjacent MAMs (Fig. 6I, arrowheads).

We next enquired whether the overexpression of non-myristoylated Stbd1 affects ER–mitochondria contact sites. For the above, we evaluated the contact surface between ER and mitochondria between untransfected control (Fig. 6J) and Stbd1(G2A)–Myc-transfected (Fig. 6K) HeLa cells. We observed that Stbd1(G2A)–Myc-transfected cells displayed a significant increase in the proportion of the mitochondrial surface that was closely associated with an ER membrane (Fig. 6K,L). At the contact sites, glycogen granules, reflecting the localization of Stbd1(G2A), were evident between the ER and the OMM (Fig. 6K). In several instances, the increase in ER–mitochondria contacts as a consequence of Stbd1(G2A) overexpression, resulted in mitochondria being almost completely enveloped by an associated ER membrane (Fig. 6K). The above findings suggest that non-myristoylated Stbd1 concentrates at MAMs, increases ER–mitochondria association, and results in mitochondrial fragmentation and clustering.

### Lack of *N*-myristoylation is not sufficient, but binding to glycogen is essential, for the targeting of Stbd1 to ER–mitochondria interfaces

**Fig. 7.**
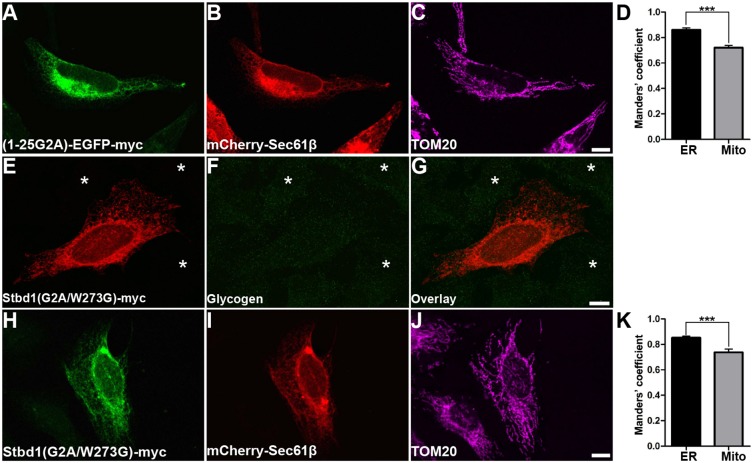
**Lack of *N*-myristoylation is not sufficient but binding to glycogen is essential for Stbd1 targeting to MAMs.** (A–D) HeLa cells co-transfected with (1-25G2A)–EGFP–Myc and mCherry–Sec61β were immunostained for Myc (A) and TOM20 (C). mCherry red fluorescence corresponding to the ER is shown in B. The (1-25G2A)–EGFP–Myc chimeric protein shows a stronger colocalization with the ER compared to mitochondria (D, thresholded Manders' coefficient, mean±s.e.m.: mitochondria, 0.720±0.018; ER, 0.861±0.015; *n*=22). (E–G) Representative images of HeLa cells transfected with Stbd1(G2A/W273G)–Myc stained for Myc (E) and glycogen (F); an overlay is shown in G. (H–K) HeLa cells transfected with Stbd1(G2A/W273G)–Myc were stained for Myc (H) and TOM20 (J). ER was labelled using mCherry–Sec61β (I). Stbd1(G2A/W273G) exhibits stronger colocalization with the ER compared to mitochondria (K, thresholded Manders' coefficient, mean±s.e.m.: mitochondria, 0.737±0.025; ER, 0.852±0.012; *n*=11). For the above, representative images are shown. Asterisks in E–G indicate untransfected cells. ***P<0.0001 (unpaired Student's *t*-test). Scale bars: 10 µm.

To gain further insight into the molecular mechanism underlying the subcellular targeting of Stbd1, we evaluated whether the lack of *N*-myristoylation alone was sufficient to promote Stbd1 localization to ER–mitochondria contact sites. For this, the first 25 amino acids of Stbd1 harbouring the G2A mutation were fused to EGFP–Myc, (1-25G2A)–EGFP–Myc, and the localization of this chimeric protein was studied in HeLa cells. (1-25G2A)–EGFP–Myc was found to coincide with the ER but only partly with mitochondria (Fig. 7A–D). This suggests that the lack of *N*-myristoylation per se is not sufficient for targeting Stbd1 to ER–mitochondria contact sites and that additional domains within the cytoplasmic tail of the protein are also required.

We next examined the importance of glycogen binding to Stbd1 in the subcellular targeting of the protein. For this, we evaluated whether a Stbd1 variant with impaired capacity to bind glycogen can be forced to ER–mitochondria contact sites. To do so, we constructed a double mutant protein variant [Stbd1(G2A/W273G)–Myc] which cannot be *N*-myristoylated and at the same time is unable to bind glycogen. Similar to Stbd1(W273G) (Fig. 2J,K), no glycogen was detected in cells transfected with the G2A/W273G double mutant (Fig. 7E–G). Assessment of the subcellular localization of the G2A/W273G variant revealed a strong colocalization with the ER (Fig. 7H–K). Moreover, as opposed to Stbd1(G2A), which retained the ability to bind glycogen (Fig. 6A–C), Stbd1(G2A/W273G) did not preferentially overlap with mitochondria (Fig. 7H–K). The above suggests that, in the absence of glycogen binding, Stbd1 is retained in the bulk ER and that association of the protein with glycogen is essential for its targeting to ER regions associated with mitochondria.

### Stbd1 silencing affects ER–mitochondria contacts and mitochondrial morphology

**Fig. 8.**
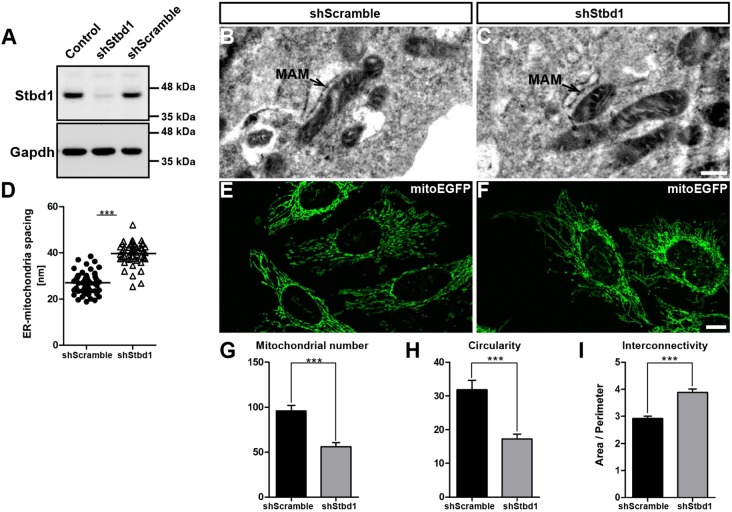
**Stbd1 silencing affects ER-mitochondrial tethering and mitochondrial morphology.** (A) Protein extracts from non-transduced controls and HeLa cells expressing either a Stbd1 shRNA or a scrambled shRNA sequence were probed with a Stbd1 antibody. An antibody against Gapdh was used as loading control. (B,C) Representative transmission electron micrographs of shScramble (B) and shStbd1 (C) cells featuring ER–mitochondria contact sites. (D) Assessment of the spacing between ER and mitochondria at ER–mitochondrial junctions between shScramble (mean±s.e.m.: 27.01±0.63 nm; *n*=53) and shStbd1 (mean±s.e.m.: 39.74±0.65 nm; *n*=52). (E,F) Representative images of the mitochondrial network in shScramble (E) and shStbd1 (F) HeLa cells labelled with mitoEGFP. (G–I) Comparison of mitochondrial morphology parameters between shScramble [mitochondria number, 96±6; circularity, 31.81±2.78; area as a ratio to the perimeter (area/perimeter), 2.92±0.08; *n*=45] and shStbd1 (mitochondria number, 56±5; circularity, 17.19±1.53; area/perimeter, 3.88±0.12; *n*=45) cells. Values represent mean±s.e.m. ***P<0.0001 (unpaired Student's *t*-test). Scale bars: 1 μm (B,C); 10 μm (E,F).

To gain insight into the role of Stbd1 in ER–mitochondria contact sites, we used a lentiviral shRNA approach to generate HeLa cells with stable knockdown of Stbd1 expression. HeLa cells expressing a scrambled shRNA sequence were generated in parallel and used as control. As evaluated by western blotting, cells expressing Stbd1-specific shRNA (shStbd1) displayed efficient silencing whereas Stbd1 protein levels in cells expressing the scrambled hairpin sequence (shScramble) were comparable to those in non-transduced controls (Fig. 8A). We next addressed the question of whether Stbd1 silencing has any effect on the spacing between ER and mitochondria at ER–mitochondria interfaces. For this, we measured the distance between ER and mitochondria at closely apposed sites in shScramble (Fig. 8B) and shStbd1 (Fig. 8C) HeLa cells on transmission electron micrographs. We found that Stbd1-knockdown cells displayed a statistically significant increase in the average distance between ER and mitochondria (mean±s.e.m.: 39.74±0.65 nm; *n*=52) as compared to shScramble control cells (mean±s.e.m.: 27.01±0.63 nm; *n*=53) (Fig. 8D), further supporting a role for Stbd1 in the physical association between ER and mitochondria and suggesting a weakening of ER–mitochondria tethers upon Stbd1 silencing.

An important function of ER–mitochondria contact sites is the regulation of mitochondrial morphology and dynamics. We therefore evaluated the effects of Stbd1 silencing on the morphology of mitochondria. For this, we first generated a construct expressing mitochondrially targeted EGFP (mitoEGFP) to label the mitochondrial network in shStbd1 and shScramble HeLa cells. Quantification of mitochondrial morphology parameters achieved by analysing confocal images of EGFP-labelled mitochondria, revealed statistically significant differences between shScramble (Fig. 8E) and shStbd1 cells (Fig. 8F). In particular, shStbd1 cells displayed reduced mitochondrial counts as compared to the controls (Fig. 8G). Furthermore, mitochondria in shStbd1 cells exhibited decreased circularity (Fig. 8H) and increased interconnectivity, indicated by an elevated area-to-perimeter ratio (Fig. 8I), compared to the shScramble control cells. The above morphological changes suggest that the mitochondrial network in Stbd1 knockdown cells consists of fewer mitochondria that were elongated and more interconnected.

## DISCUSSION

In the present study, we provide evidence that Stbd1 is an ER-resident transmembrane protein that also localizes to MAMs, and identify its N-terminal hydrophobic region as a signal-anchor sequence being both necessary and sufficient to promote targeting and retention of the protein to the ER membrane. We further demonstrate that mouse Stbd1 is able to tether ER membranes resulting in the formation of OSER in HeLa cells. Although, OSER formation has been observed in a variety of cells and species, both under physiological and pathological conditions, the biological significance of its formation remains elusive. OSER was shown to be induced by the overexpression of a number of ER-resident proteins, anchored through a transmembrane domain and capable of undergoing weak homotypic interactions through their cytoplasmic tail (Snapp et al., 2003). Stbd1 shares similar features with these proteins since it is an integral ER membrane protein, anchored through its N-terminal hydrophobic region and projecting into the cytosol. Moreover, human Stbd1 was reported to form dimers through its C-terminal CBM20 domain (Jiang et al., 2010). Importantly, OSER-like structures that stained positive for Stbd1 and glycogen, were observed endogenously in C2C12 mouse myoblasts. This suggests that ER rearrangement and glycogen recruitment to organized ER membranes is an intrinsic property of Stbd1 that also occurs under endogenous levels of expression.

We demonstrate that mouse Stbd1 is *N*-myristoylated and that this lipid modification significantly affects the subcellular localization of the protein. Whereas myristoylated Stbd1 is preferentially retained in bulk ER, inhibition of *N*-myristoylation favours its localization to MAMs in HeLa cells. It is well established that *N*-myristoylation mainly occurs on cytoplasmic proteins, and only very few eukaryotic integral membrane proteins were found to be *N*-myristoylated. These include the mammalian NADH-cytochrome b(5) reductase (Cyb5R3) and the dihydroceramide Δ4 desaturase 1 (DES1, also known as DEGS1), which are targeted to both the ER and mitochondria. In both Cyb5R3 and DES1, *N*-myristoylation favours their localization to mitochondria (Borgese et al., 1996; Beauchamp et al., 2009). As demonstrated for Cyb5R3, *N*-myristoylation interferes with the binding of the signal recognition particle to the nascent N-terminal domain of the protein preventing ER targeting and enabling mitochondrial localization (Colombo et al., 2005). On the other hand, *N*-myristoylation of the integral membrane protein Lunapark did not affect its targeting to the ER membrane (Moriya et al., 2013). Our data indicate that the latter also applies for Stbd1, since targeting of the protein to the ER occurs independently of *N*-myristoylation suggesting that the above lipid modification does not generally interfere with ER targeting of integral membrane proteins.

Palmitoylation, an alternative type of lipid modification, was shown to promote the enrichment of the transmembrane ER proteins calnexin and thioredoxin-related oxidoreductase (TMX, also known as TMX1) in MAMs (Lynes et al., 2012). This raised the hypothesis that lipid modifications could serve as a mechanism to target ER proteins to MAMs (Vance, 2014). In contrast to the reported examples of calnexin and TMX, targeting of Stbd1 to MAMs was promoted by the lack of lipidation. Stbd1 therefore constitutes a unique case, since to our knowledge a similar localization of an ER protein in MAMs as a consequence of the absence of a lipid modification has not been previously reported.

Our data imply the existence of two intracellular pools of Stbd1 – an *N*-myristoylated pool and a non-myristoylated pool that are preferentially localized to the ER and MAMs, respectively. Although *N*-myristoylation is generally considered an irreversible lipid modification, the existence of non-myristoylated protein substrates has been reported *in vivo* (McIlhinney and McGlone, 1990). However, the molecular mechanisms underlying the generation of these non-myristoylated pools are not clear. How could the presence or absence of myristate promote localization of Stbd1 to bulk ER or MAMs, respectively? The above could involve a mechanism similar to the one reported for the mammalian Golgi reassembly stacking proteins (GRASPs), which, although they are not integral membrane proteins, are anchored to membranes by an N-terminal myristic acid and interaction with a membrane-bound receptor. As demonstrated for the GRASP domain, *N*-myristoylation restricts its orientation on the membrane, thus favouring trans-pairing and membrane tethering, whereas non-myristoylated GRASPs lack a fixed orientation and inefficiently tether membranes (Heinrich et al., 2014). Accordingly, *N*-myristoylation may lock the N-terminal transmembrane domain of Stbd1 in a fixed orientation in the ER membrane thus promoting trans-dimerization and membrane tethering resulting in the formation of OSER whereas lack of myristate may compromise dimerization enabling the localization of the protein to MAMs. Interestingly, and in support of the above, the herein reported G2A/W273G Stbd1 double mutant, in which *N*-myristoylation and glycogen binding are simultaneously abolished and is therefore retained in bulk ER, does not induce the formation of OSER structures (Fig. 7E) in contrast to the single Stbd1(W273G) mutant (Fig. 2J,L). The selective association of non-myristoylated Stbd1 with MAMs could be related to their unique lipid composition. In contrast to membranes of bulk ER, MAMs are enriched in cholesterol and sphingolipids which may determine their specific association with proteins, as demonstrated for the Sigma-1 receptor (Hayashi and Fujimoto, 2010). Non-myristoylated Stbd1 may thus preferentially associate with lipids present in MAMs whereas modification of the N-terminal transmembrane domain through the addition of myristate may interfere with the above, resulting in Stbd1 localization to bulk ER.

Our findings further indicate that the absence of *N*-myristoylation per se is not sufficient to promote Stbd1 localization to MAMs and that this can only occur when glycogen is bound to the protein. Glycogen is therefore an important determinant of Stbd1 targeting and might mediate the interaction between Stbd1 and other proteins present at ER–mitochondria contact sites. Considering the proposed role for Stbd1 as a selective autophagy receptor for glycogen, our findings indicate that, depending on its myristoylation status, Stbd1 can recruit glycogen to OSER and ER–mitochondria contact sites. Interestingly, both subcellular domains have been associated with the process of autophagy. OSER structures were reported to be sequestered into LC3-positive autophagosomes (Lingwood et al., 2009) whereas ER–mitochondria contact sites have recently been identified as the site of autophagosome formation (Hamasaki et al., 2013). It is thus conceivable that Stbd1-mediated recruitment of glycogen to OSER structures and ER–mitochondria contact sites may represent alternative means through which glycogen is selectively sequestered into autophagosomes and targeted for lysosomal degradation.

ER–mitochondria contact sites have gained a lot of attention recently, and accumulated evidence suggests an important role for these attachment sites in a variety of cellular processes. These include, in addition to their aforementioned involvement in the initiation of the autophagic process, the physical tethering between ER and mitochondria, mitochondrial dynamics, and the regulation of mitochondrial morphology and function, as well as the transport of Ca^2+^ and lipids from the ER to mitochondria (Marchi et al., 2014; Vance, 2014). Our data uncover a new role for Stbd1 in the physical coupling between ER and mitochondria. This is supported by the increase of the contact surface between ER and mitochondria as a result of the forced targeting of the protein to MAMs. On the other hand, shRNA-mediated knockdown of Stbd1 in HeLa cells resulted in an increase of the spacing between ER and mitochondria. Importantly, both the overexpression of Stbd1(G2A) and the silencing of endogenous Stbd1 affected the morphology of the mitochondrial network. While the overexpression of MAM-targeted Stbd1 caused profound mitochondrial fragmentation and clustering, Stbd1 knockdown resulted in morphological alterations consistent with increased mitochondrial connectivity.

The molecular details of the physical attachment between ER and mitochondria remain largely unknown but are best studied in yeast in which ER–mitochondrial tethering has been shown to be mediated by protein complexes such as the ER–mitochondria encounter structure (ERMES) (Kornmann et al., 2009) and the ER membrane protein complex (EMC) (Lahiri et al., 2014). In mammals, proteins regulating ER–mitochondria juxtaposition include the phosphofurin acidic cluster protein 2 (PACS2) (Simmen et al., 2005), the VAPB–PTPIP51 (PTPIP51 is also known as RMDN3) (Stoica et al., 2014) and ITPR1–Grp75–VDAC1 (Grp75 is also known as HSPA9) complexes (Szabadkai et al., 2006) and mitofusin 2 (Mfn2), a GTPase involved in mitochondrial fusion with probably the strongest implication in ER–mitochondria coupling (de Brito and Scorrano, 2008). The role of Mfn2 in promoting ER–mitochondria tethering is supported by different studies (Sebastian et al., 2012; Chen et al., 2012; Schneeberger et al., 2013; Hailey et al., 2010; Hamasaki et al., 2013; Area-Gomez et al., 2012) and has been recently re-confirmed (Naon et al., 2016) following the report of contradicting findings suggesting that Mfn2 ablation instead strengthens ER–mitochondria coupling (Cosson et al., 2012; Filadi et al., 2015). Stbd1 could be implicated in ER–mitochondria association by regulating the aforementioned ER–mitochondria tethers or by interacting with an as yet unidentified partner on the OMM.

Interestingly, although Mfn2 is involved in mitochondrial fusion, its overexpression causes fission-mediated fragmentation and clustering of mitochondria (Huang et al., 2007), an effect similar to that upon the overexpression of non-myristoylated Stbd1. In the case of Mfn2, the molecular basis of the above is not clear. Data presented here indicate that fragmentation and clustering of mitochondria upon Stbd1(G2A) overexpression is accompanied by the increase of the ER–mitochondria contact surface. Nevertheless, the increased coupling between the ER and mitochondria, as such, probably does not underlie the above mitochondrial phenotype. This is supported by the fact that overexpression of the ER-resident protein VAPB and its interacting partner, the OMM protein PTPIP51, resulted in a similar increase of ER–mitochondria contacts, yet mitochondrial morphology was not affected (Stoica et al., 2014). It is thus possible that Stbd1 interacts with proteins promoting mitochondrial fission or inhibiting fusion and recruits them to ER–mitochondria junctions. A number of proteins [Mff, Fis1, MiD49 (also known as MIEF2) and MiD51 (also known as MIEF1)] function as adaptors that either cooperatively or independently recruit the dynamin-related protein Drp1 (also known as DNM1L) to sites of mitochondrial fission (Loson et al., 2013; Osellame et al., 2016). Stbd1 might serve a similar role by interacting either directly with Drp1 or in complex with the known adaptor proteins. The above hypothesis would explain the observed shift of the equilibrium between mitochondrial fusion and fission towards fusion upon Stbd1 silencing.

An intriguing, but still unresolved question at this stage, concerns the role of glycogen bound to Stbd1 and whether it is either directly or indirectly implicated in ER–mitochondria tethering, and in the balance between mitochondrial fusion and fission. A number of studies have established a link between mitochondrial dynamics and nutrient status. Lack of nutrients was shown to correlate with mitochondrial elongation, whereas nutrient excess was associated with fragmented mitochondrial morphology (Molina et al., 2009; Gomes et al., 2011; Jheng et al., 2012). These changes enable the cell to adjust ATP production in response to nutrient supply. Our finding that targeting of Stbd1 to MAMs requires its binding to glycogen raises the intriguing hypothesis that Stbd1-mediated glycogen recruitment to MAMs may represent a mechanism that signals nutrient status to mitochondria and accordingly influences their morphology.

## MATERIALS AND METHODS

### Expression constructs

Details and schematic representation of the expression constructs used in this study are shown in Table S1 and Fig. S4.

### Cell culture and transfections

HeLa, C2C12 and HEK293T cells were obtained from the American Type Culture Collection (ATCC), cultured in high-glucose Dulbecco's modified Eagle's medium (DMEM; Biosera) supplemented with 10% fetal bovine serum (FBS; Biosera), 1× penicillin-streptomycin (Biosera) and 1× GlutaMAX (GIBCO) and incubated at 37°C under 5% CO_2_. All cell lines were tested and found to be free of mycoplasma contamination. Transfections were performed using either Lipofectamine^®^ LTX with PLUS™ Reagent (Invitrogen) following the manufacturer's instructions or calcium phosphate co-precipitation.

### Antibodies

Antibodies used in the study are listed in Table S2.

### Immunofluorescence staining and microscopy

Cells grown on glass coverslips were fixed with 4% paraformaldehyde (PFA) in PBS or methanol for 10 min at room temperature or −20°C, respectively. PFA-fixed cells were permeabilized with 0.1% Triton X-100 in PBS for 10 min at room temperature. Blocking was performed with 5% normal goat serum (Biosera) in PBS with 0.05% Tween 20 (PBST). Cells were incubated with primary antibodies diluted in 5% goat serum in PBST overnight at 4°C and with appropriate secondary fluorescent antibodies for 1 h at room temperature. Nuclei were counterstained with DAPI and coverslips mounted with Mowiol. Images were obtained on a TCSL confocal microscope (Leica), using a 40× or 63× oil-immersion objective lens and 2.3–3× digital zoom.

### Protein preparation from cell lysates and cell culture supernatants

Following transfection, cells were cultured for 24 h in high-glucose DMEM supplemented with low (1%) FBS. Cell culture supernatants were collected and cells lysed in 150 mM NaCl, 1% Triton X-100, 0.1% SDS, 50 mM Tris-HCl pH 8.0 for 30 min on ice and centrifuged at 13,000* **g*** for 15 min. Proteins from culture supernatants were precipitated, by means of trichloroacetic acid-acetone precipitation, resuspended in 1× alkaline SDS-PAGE buffer (50 mM Tris-HCl pH 8.0, 2% SDS, 100 mM DTT, 10% glycerol) and analyzed by western blotting. For the evaluation of Stbd1 silencing, shStbd1 and shScramble cells cultured in DMEM with 10% FBS were lysed as above.

### Subcellular fractionation

Microsomal, mitochondrial and MAM fractions were isolated from HeLa cells according to a previously published procedure (Bozidis et al., 2007). Briefly, cells were homogenized in ice-cold sucrose homogenization medium (0.25 M sucrose, 10 mM HEPES, pH 7.4) in a Potter–Elvehjem homogenizer. The homogenate was centrifuged twice at 600* **g*** for 5 min at 4°C to remove nuclei, cell debris and unbroken cells, and mitochondria plus MAMs were pelleted by centrifugation at 10,300* **g*** for 10 min at 4°C. Microsomes were pelleted from the supernatant by ultracentrifugation (Optima L-100 XP, Beckman Coulter) at 100,000* **g*** for 1 h at 4°C. The mitochondria plus MAM pellet was resuspended in ice-cold mannitol buffer A (0.25 M mannitol, 0.5 M EGTA, 5 mM HEPES, pH 7.4), layered on top of 10 ml of ice-cold 30% Percoll (Sigma-Aldrich) solution [1 volume 90% stock isotonic Percoll (9 volumes Percoll and 1 volume 0.25 M sucrose), 2 volumes mannitol buffer B pH 7.4 (0.225 M mannitol, 1 mM EGTA, 25 mM HEPES)] and ultracentrifuged at 95,000* **g*** for 65 min at 4°C. The MAMs and the multiband mitochondrial fractions were recovered from the Percoll gradient using a 1 ml syringe and a 20G needle. Mitochondria were pelleted by centrifugation at 6300* **g*** for 10 min at 4°C whereas the MAM fraction was recovered by ultracentrifugation at 100,000* **g*** for 1 h at 4°C.

### Western blotting

Proteins were resolved on a 12% SDS-PAGE gel and transferred onto a nitrocellulose membrane (Porablot NCP, Macherey-Nagel). Following blocking with 5% non-fat milk in TBST (0.01 M Tris-HCl pH 8.0, 150 mM NaCl, 0.05% Tween 20), the membrane was sequentially incubated with the primary and HRP-conjugated secondary antibodies diluted in blocking buffer. Proteins were detected using chemiluminescent substrates (Amersham™ ECL™ Select Western Blotting Detection Reagent). Images were obtained with a BioSpectrum 810 imaging system (UVP). For an estimation of the amount of protein loaded, membranes were either probed with an antibody against Gapdh or stained with amido black staining solution (Sigma-Aldrich) after development, according to the manufacturer's instructions.

### YnMyr labelling and myristoylation assay

The myristoylation assay was performed according to a previously published procedure (Thinon et al., 2014). Briefly, HeLa cells were transfected with Stdb1–Myc or Stdb1(G2A)–Myc expression plasmids, or the empty vector, cultured in the presence of the NMT inhibitor DDD856462 (Alibhai et al., 2013; Thinon et al., 2014) (5 μM) or vehicle (DMSO) and treated with YnMyr. Cells were subsequently lysed and incubated with a click mixture consisting of 0.1 mM azido-TAMRA-PEG-Biotin (AzTB) (Heal et al., 2012), 1 mM CuSO_4_, 1 mM tris(2-carboxyethyl)phosphine (TCEP) and 0.1 mM tris(benzyltriazolylmethyl)amine (TBTA). Labelled proteins were pulled down with Dynabeads MyOne Streptavidin C1 (Invitrogen). Protein samples (‘input’, ‘unbound’ and ‘pulldown’) were separated on a 12% SDS-PAGE gel. In-gel fluorescence was detected using a Typhoon FLA 9500 machine (GE Healthcare). Stbd1–Myc and Stbd1(G2A)–Myc were detected by western blotting using an antibody against Myc (Millipore). An anti-HSP90 antibody was used as loading and negative control.

### Transmission electron microscopy

Cells were pelleted, fixed with 2.5% glutaraldehyde in 0.1 M phosphate buffer, pH 7.2, for 24 h at 4°C and washed with 0.1 M phosphate buffer. 1% melted agar (Sigma-Aldrich) was added to the pelleted cells and placed at −20°C for 5 min. The solidified cell pellet–agar block was post-fixed with 1% osmium tetroxide, dehydrated in a graded ethanol series, cleared in propylene oxide and embedded in an epon and araldite resin mixture (Agar Scientific). Ultrathin (80 nm) sections were prepared on a Reichert UCT ultramicrotome (Leica). Sections with a silver-gold interference colour were mounted on 200 mesh copper grids (Agar Scientific) and contrasted with uranyl acetate and lead citrate. Images were obtained on a JEM 1010 transmission electron microscope (JEOL) equipped with a Mega View III digital camera (Olympus).

### shRNA-mediated Stbd1 silencing

The following oligonucleotides containing a short hairpin for Stbd1 (shStbd1) were annealed and cloned in pLKO.1-TRC (Addgene plasmid #10878, deposited by David Root) (Moffat et al., 2006), at *AgeI-EcoRI* restriction sites: forward, 5′-CCGGGAAGAATGCAGCAATAGATTCCTCGAGGAATCTATTGCTGCATTCTTCTTTTTG-3′; reverse, 5′-AATTCAAAAAGAAGAATGCAGCAATAGATTCCTCGAGGAATCTATTGCTGCATTCTTC-3′. A pLKO.1-scramble shRNA vector (Addgene plasmid #1864, deposited by David Sabatini) (Sarbassov et al., 2005), was used as control. Lentiviral particles were produced in HEK293T cells following co-transfection of shStbd1 or shScramble vector with the accessory vectors pMD2.G and psPAX2 (Addgene plasmids #12259 and #12260, respectively, deposited by Didier Trono). The cell culture supernatant containing lentiviral particles was used to infect HeLa cells. Finally, cells stably expressing shStbd1 or shScramble were selected with puromycin (3 μg/ml). Efficiency of gene silencing was evaluated by western blotting using a Stbd1-specific antibody.

### Image analysis

Mitochondrial morphology parameters of cells either transiently transfected with mitoEGFP or immunostained with TOM20 were quantified on confocal images using the Mito-morphology macro of ImageJ (Dagda et al., 2009). Quantification of colocalization (thresholded Manders’ coefficients; Manders et al., 1993) was performed on confocal images using the Coloc 2 plugin of the Fiji software.
